# Prognostic Value of the CALLY Index in Diffuse Large B-Cell Lymphoma: Linking Inflammation, Nutrition, and Tumor Biology

**DOI:** 10.3390/cancers18050846

**Published:** 2026-03-05

**Authors:** Zorica Cvetković, Ilija Bukurecki, Snežana Pejić, Anica Divac Pravdić, Miroslav Pavlović, Vesna Vučić, Olivera Marković

**Affiliations:** 1 Department of Hematology, University Hospital Medical Center Zemun, 11080 Belgrade, Serbia; 2 Faculty of Medicine, University of Belgrade, 11000 Belgrade, Serbia; 3 Department of Hematology, University Hospital Medical Center Bežanijska Kosa, 11080 Belgrade, Serbia; 4 Department of Molecular Biology and Endocrinology, Institute of Nuclear Sciences, National Institute of the Republic of Serbia, University of Belgrade, 11000 Belgrade, Serbia; 5 Department of Hematology, University Hospital Medical Center Zvezdara, 11000 Belgrade, Serbia; 6 Center of Research Excellence in Nutrition and Metabolism, Institute for Medical Research, National Institute of the Republic of Serbia, University of Belgrade, 11000 Belgrade, Serbia

**Keywords:** diffuse large B-cell lymphoma (DLBCL), CALLY index, prognosis, relapsed/refractory (R/R), biomarker

## Abstract

**Background/Objectives:** Diffuse large B-cell lymphoma (DLBCL) is the most common subtype of non-Hodgkin lymphoma and remains incurable in approximately 30–40% of patients despite advances in immunochemotherapy. Although gene expression profiling has improved risk stratification, there is an ongoing need for non-invasive, cost-effective, and clinically practical biomarkers to identify patients at high risk of treatment resistance or relapse (R/R). Systemic inflammation plays a pivotal role in DLBCL pathogenesis, impacting both tumor progression and treatment response. The C-reactive protein–albumin–lymphocyte (CALLY) index, integrating markers of inflammation, nutritional status, and immune competence, has demonstrated prognostic relevance in solid tumors; however, its relevance in hematologic malignancies remains unexplored. **Methods**: We retrospectively analyzed 180 adults with newly diagnosed DLBCL, NOS (not otherwise specified) who received frontline rituximab-based immunochemotherapy (R-CHOP or CHOP-like regimens) between January 2014 and December 2019 at three tertiary centers in Serbia. The median age was 67 years (IQR 59–73), and 56.1% were female. Receiver operating characteristic (ROC) analysis determined 6.5 as the optimal CALLY index cut-off (AUC 0.744, 95% CI 0.670–0.817; *p* < 0.001). **Results**: A low CALLY index (<6.5) was significantly associated with adverse clinical features, including anemia, elevated lactate dehydrogenase and β2-microglobulin, poor ECOG performance status, bulky disease, advanced stage, and unfavorable IPI, R-IPI, and NCCN-IPI scores (all *p* < 0.001). In contrast, no associations were observed with tumor subtype, immunophenotype, or comorbidities. Furthermore, patients with CALLY <6.5 showed lower overall response rates to treatment (59.6% vs. 85.5%, *p* < 0.001) and higher relapse rates (21.0% vs. 6.2%, *p* = 0.014). They also experienced reduced 3- and 5-year overall survival (OS) and event-free survival (EFS) (all *p* < 0.001). In multivariate analysis, a low CALLY index independently predicted poorer OS (HR 2.04, 95% CI 1.13–3.67; *p* = 0.017) and EFS (HR 1.89, 95% CI 1.13–3.14; *p* = 0.015). In addition, it independently identified patients at risk of relapsed/refractory (R/R) disease (OR 2.50, 95% CI 1.02–10.10; *p* = 0.04), outperforming standard prognostic indices. **Conclusions**: The CALLY index is a simple, low-cost, and widely accessible biomarker that independently predicts prognosis in DLBCL, NOS. It outperforms standard indices in identifying R/R cases. The CALLY index may enhance risk stratification and guide individualized treatment strategies.

## Simple Summary

Diffuse large B-cell lymphoma (DLBCL) is the most common type of non-Hodgkin lymphoma, but many patients experience relapse or resistance to standard frontline treatment. Better and easily available biomarkers are needed to improve risk assessment. The C-reactive protein–albumin–lymphocyte (CALLY) index is a simple score based on blood markers reflecting inflammation, nutritional status, and immune function. While the CALLY index has shown prognostic value in solid tumors, its relevance in hematologic malignancies remains unexplored. In this multicenter retrospective study, we analyzed 180 newly diagnosed DLBCL, NOS (not otherwise specified) patients treated with rituximab-based immunochemotherapy. A low CALLY index at diagnosis was independently associated with inferior overall and event-free survival, and a higher risk of refractory or relapsed disease, independent of established prognostic scores. Our findings suggest that the CALLY index is an inexpensive, accessible tool that may help identify high-risk patients and guide more personalized treatment strategies in DLBCL.

## 1. Introduction

Diffuse large B-cell lymphoma (DLBCL) is the most common and aggressive subtype of non-Hodgkin lymphoma (NHL) [1]. The disease primarily affects individuals in their mid-to-late 60 s, with an annual incidence of 5–7 cases per 100,000 people in Western countries [2]. Due to demographic shifts and an aging global population, the incidence of DLBCL is expected to rise further in the coming years [3,4]. Despite significant advances in targeted therapies, approximately 30–40% of patients eventually experience relapse or develop refractory disease (R/R) [5]. Early identification of high-risk patients is therefore crucial for optimizing treatment strategies and improving outcomes, highlighting the ongoing need for reliable prognostic biomarkers [6,7,8].

Conventional prognostic models, including the International Prognostic Index (IPI), Revised IPI (R-IPI), and NCCN-IPI, stratify patients using clinical parameters such as age, performance status (PS), lactate dehydrogenase (LDH) levels, clinical stage (CS), and extranodal (EN) involvement [9,10,11]. While effective at a population level, these indices often fail to identify subgroups of patients who will experience treatment failure or relapse after initially responding to frontline therapy [12]. Furthermore, these models do not adequately account for host-related factors such as the tumor microenvironment, systemic inflammation, nutritional status, and immune competence, all of which are increasingly recognized as critical determinants of cancer progression and treatment outcomes [13,14,15,16,17,18].

Biomarkers related to inflammation, nutrition, and immune competence, such as C-reactive protein (CRP), serum albumin, and absolute lymphocyte count (ALC), have been individually associated with survival in DLBCL [19,20,21,22]. Elevated CRP is a well-established marker of systemic inflammation triggered by tumor-induced cytokines [23,24]. Hypoalbuminemia indicates malnutrition and a catabolic state driven by chronic inflammation [25,26], while lymphopenia suggests impaired immune surveillance [27,28]. However, each marker can be influenced by comorbidities, limiting its specificity for lymphoma prognosis.

To enhance prognostic accuracy, composite indices that integrate these parameters have been developed. The CALLY index, calculated as (Serum Albumin × ALC)/(CRP × 10^4^), was initially proposed for hepatocellular carcinoma [29] and has since been validated in several solid tumors [20,21,22,23,24,25,26,27,28,29,30,31,32,33,34]. However, evidence supporting its prognostic relevance in hematologic malignancies remains limited, including DLBCL. To our knowledge, our team was the first to present preliminary data suggesting the CALLY index as a promising prognostic tool in DLBCL [35], and similar findings have only recently been reported on an independent Turkish cohort [36].

In this context, the present study aims to systematically evaluate the prognostic significance of the CALLY index in a homogeneous cohort of patients with newly diagnosed DLBCL, not otherwise specified (NOS), treated with standard rituximab-based immunochemotherapy (ICT). We hypothesize that the CALLY index will outperform its individual components and provide incremental prognostic value beyond conventional prognostic models. Furthermore, we investigate whether the CALLY index reflects lymphoma-specific biology, rather than nonspecific factors such as baseline health status or comorbidity burden.

## 2. Materials and Methods

This study was conducted in accordance with the ethical principles of the Declaration of Helsinki (1975, as revised in 2013) and received approval from the Institutional Ethics Committee (Approval No. 92/1, dated 15 October 2024). The need for informed consent was waived due to the retrospective design, absence of direct patient contact, and the use of anonymized, de-identified data for analysis.

### 2.1. Study Population

We retrospectively reviewed the medical records of 180 consecutive, newly diagnosed adult patients with DLBCL, not otherwise specified (DLBCL, NOS), who had complete and relevant clinical, laboratory, and histopathological data. Patients with high-grade B-cell lymphomas, other large B-cell lymphoma subtypes, or concurrent malignancies were excluded. In addition, 19 MYC-positive cases were excluded due to the absence of fluorescence in situ hybridization (FISH) analysis, which precluded accurate molecular classification. None of the patients presented with the leukemic phase of lymphoma at the time of diagnosis. All included patients were diagnosed and treated from January 2014 to December 2019 at three tertiary university hospitals in Belgrade, Serbia: University Hospital Medical Center Zemun, University Hospital Medical Center Bežanijska kosa, and University Hospital Medical Center Zvezdara.

Baseline demographic data included age and sex. An age cut-off of >60 years was used to define adverse prognosis, consistent with established prognostic indices [9,10,11]. PS was assessed using the Eastern Cooperative Oncology Group (ECOG) scale [37]. The presence and severity of comorbidities, including prior malignancies in remission, were evaluated using the Cumulative Illness Rating Scale (CIRS) [38], with a score >6 considered clinically significant based on its association with inferior survival in lymphoid malignancies [39,40].

### 2.2. Laboratory Analyses

At diagnosis, the following laboratory cut-off values were applied: hemoglobin (Hb) <100 g/L [41]; ALC <1 × 10^9^/L [42]; absolute monocyte count (AMC) >0.63 × 10^9^/L [43]; and platelet count <157 × 10^9^/L [44]. CRP (mg/L), LDH (U/L), and β2-microglobulin (β2M, mg/L) were defined as elevated when exceeding the upper limit of normal (ULN). Hypoalbuminemia was defined as serum albumin <35 g/L [45].

The CALLY index was calculated as follows: CALLY = (serum albumin × ALC)/(CRP × 10^4^).

### 2.3. Histopathology and Clinical DLBCL-Related Characteristics

The diagnosis of DLBCL, NOS was established according to the 5th edition of the World Health Organization Classification of Haematolymphoid Tumours (WHO-HAEM5) [46]. Cases were further categorized by cell of origin (COO) into germinal center B-cell-like (GCB) or non-GCB subtypes using the Hans immunohistochemical algorithm [47]. The expression of BCL2 and BCL6 was documented, and a high proliferative index was defined as Ki-67 >70% [48].

Clinical features included EN involvement and bulky disease, which was defined as a tumor mass >10 cm on baseline imaging [49]. Staging was determined according to the Ann Arbor system, with CS I–II classified as limited disease and CS III–IV as advanced disease [50].

Risk stratification was performed using the IPI [9], R-IPI [10], and NCCN-IPI [11].

### 2.4. Treatment and Response Assessment

All patients received first-line treatment with R-CHOP (rituximab, cyclophosphamide, doxorubicin, vincristine, and prednisone) or an R-CHOP–like ICT regimen, in accordance with institutional treatment guidelines. The R-mini-CHOP regimen was administered to 8 patients older than 80 years, while 14 anthracycline-ineligible patients received the R-COEP (rituximab, cyclophosphamide, etoposide, vincristine, and prednisone) regimen. Treatment response was evaluated using the Lugano classification criteria [51]. The overall response rate (ORR) was defined as the proportion of patients achieving complete remission (CR) or partial remission (PR). Final follow-up, including assessment of R/R disease, was completed in December 2024. Overall survival (OS) was calculated from the date of diagnosis to death from any cause. Event-free survival (EFS) was defined as the time from initiation of ICT until relapse or progression, unplanned retreatment after initial ICT, death from any cause, or last follow-up [52]. Patients who were alive and event-free at the last follow-up were censored.

### 2.5. Statistical Analysis

Statistical analyses were performed using IBM SPSS Statistics (Version 27). Categorical variables were summarized as frequencies and percentages, with associations assessed via chi-square tests. Survival probabilities for OS and EFS were estimated using the Kaplan–Meier method, and group differences were evaluated with the log-rank test. The optimal cutoff value for the CALLY index was determined using time-dependent ROC curve analysis to account for censored survival data. Independent prognostic factors for OS and EFS were identified using Cox proportional hazards regression models, with results expressed as hazard ratios (HRs) and 95% confidence intervals (CIs).

Multivariate logistic regression was used to identify independent predictors of treatment response (ORR) and relapsed/refractory (R/R) status, with results expressed as odds ratios (ORs) and 95% CIs. All multivariate models were constructed using a clinically guided, parsimonious approach. To mitigate multicollinearity and ensure model stability, composite scoring systems (NCCN-IPI and CALLY index) were utilized as consolidated markers; their individual constituent components (e.g., age, performance status, and laboratory markers) were therefore excluded from the multivariate models to avoid data redundancy.

Furthermore, while the IPI, R-IPI, and NCCN-IPI were each significant in univariate assessments, they exhibited considerable multicollinearity. Consequently, only the NCCN-IPI was retained for the final multivariate analysis as it demonstrated the greatest statistical stability and represents the most contemporary scoring system. To address potential bias and ensure the reproducibility of our findings, logistic regression models were further validated via internal bootstrapping (1000 samples). A *p*-value < 0.05 was considered statistically significant.

## 3. Results

**Figure 1:**
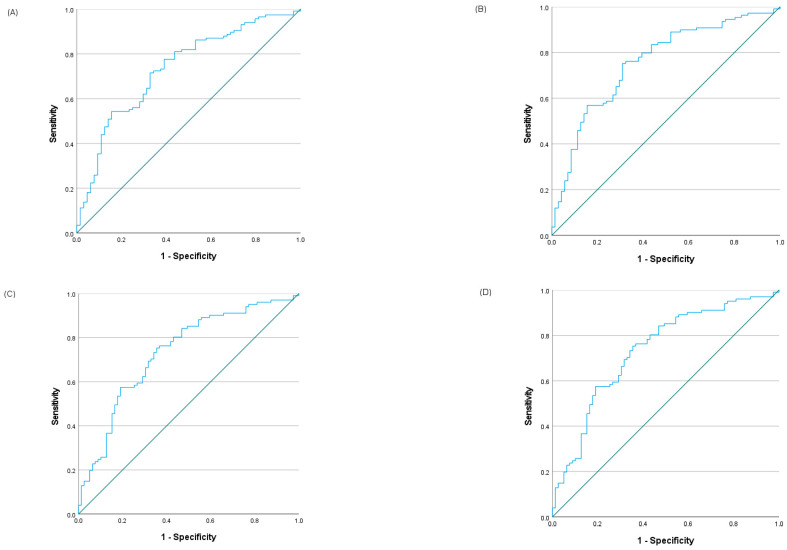
Time-dependent ROC curves of the CALLY index for predicting survival in patients with DLBCL, NOS at multiple time points: (**A**) 2 years, (**B**) 3 years, (**C**) 5 years, and (**D**) 10 years.

The time-dependent ROC analysis yielded consistently high AUC values across all evaluated time points (2, 3, 5, and 10 years), ranging from 0.733 to 0.755 (95% CI: 0.670–0.817; *p* < 0.001; Supplementary Table S1). The optimal threshold for the CALLY index was defined by the maximal combined sensitivity and specificity across these intervals, with values converging between 6.41 and 6.59 (Figure 1). Based on these findings, a unified cutoff of 6.5 was established, which effectively stratified the cohort into low CALLY (<6.5; 57.8%) and high CALLY (≥6.5; 42.2%) groups.

### 3.1. Clinicopathological Characteristics and the CALLY Index

The median age of the study population was 67 years (IQR: 59–73), with a female predominance (F:M = 1.29). Age distribution did not differ significantly between CALLY groups, but the proportion of male patients was higher in the CALLY ≥6.5 group compared to the CALLY <6.5 group (53.9% vs. 36.5%; *p* = 0.02). Poor ECOG PS (≥2) was observed in 22.8% of the total cohort and was significantly more common among patients with a CALLY index <6.5 (33.7% vs. 7.49; *p* < 0.001). The prevalence and severity of comorbidities, as measured by the CIRS, were similar between groups.

A low CALLY index was significantly associated with anemia (26% vs. 5.3%; *p* < 0.001), lymphopenia (38.5% vs. 14.5%; *p* < 0.001), elevated LDH levels (74% vs. 35.5%; *p* < 0.001), hypoalbuminemia (43.3% vs. 3.9%; *p* < 0.001), elevated β2M levels (85.6% vs. 42.1%; *p* < 0.001), and elevated CRP (96.2% vs. 27.6%; *p* < 0.001). The non-GCB histological subtype was more prevalent than GCB (66.7% vs. 33.3%), with no significant differences in distribution between CALLY groups (*p* = 0.915). The expression of BCL2, BCL6, MUM1, as well as a high Ki-67 proliferative index, did not differ significantly between the groups (*p* > 0.05).

Bulky disease was more frequent in the CALLY <6.5 group (22.1% vs. 2.6%; *p* < 0.001), while EN involvement was documented in 45% of patients, with no difference between groups. Advanced disease (Ann Arbor CS III/IV) was also significantly more common among patients with a CALLY index <6.5 (78.8% vs. 42.1%; *p* < 0.001). Patients with a low CALLY index were significantly more likely to have high IPI (65.4% vs. 19.7%; *p* < 0.001), poor R-IPI (62.5% vs. 13.2%; *p* < 0.001), and high-risk NCCN-IPI (79.8% vs. 44.7%; *p* < 0.001) scores (Table 1).

### 3.2. Patient Follow-Up and Survival Analysis

Median follow-up for the entire cohort was 63 months, and at the end of follow-up, 56.1% of patients were alive. Of the total cohort, 18 of 79 patients (22.8%) died from non-DLBCL-related causes. The most common causes were cardiovascular events, including 4 heart failures, 2 acute myocardial infarctions, and 3 ischemic strokes. This was followed by deaths from COVID-19 infection (5 patients). Additionally, two patients died from secondary malignancies (prostate and lung cancer), and one patient died in a car accident. Notably, non-DLBCL-related deaths occurred more frequently in the CALLY ≥6.5 group, although this difference did not reach statistical significance (38.9% vs. 18.0%; *p* = 0.064).

**Figure 2:**
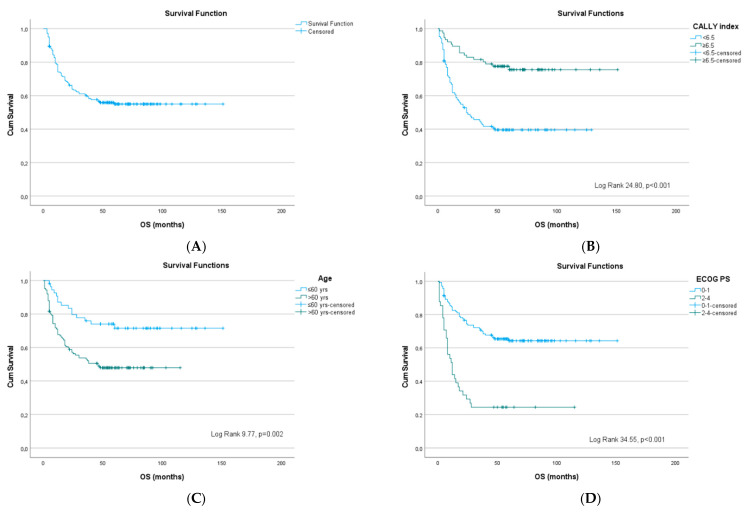

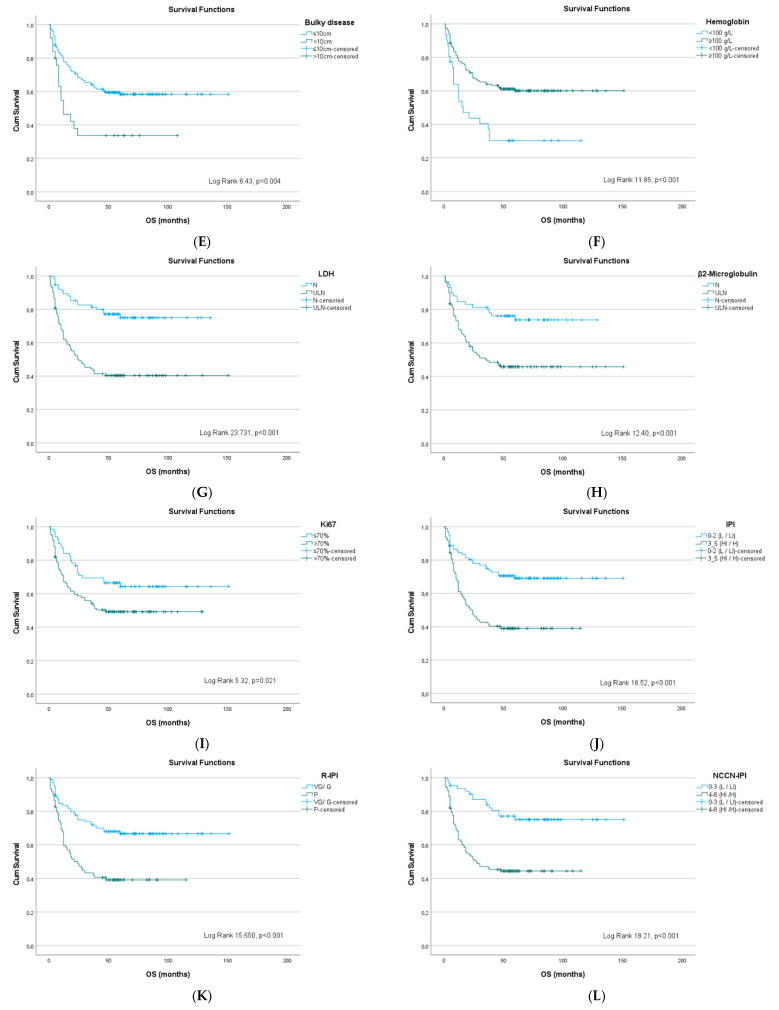
Kaplan–Meier curves for overall survival in patients with DLBCL, NOS: (**A**) entire cohort, stratified by (**B**) CALLY index, (**C**) age, (**D**) ECOG PS, (**E**) bulky disease, (**F**) hemoglobin level, (**G**) lactate dehydrogenase, (**H**) β2-microglobulin, (**I**) Ki67, (**J**) IPI, (**K**) R-IPI, and (**L**) NCCN-IPI.

Survival was significantly lower among patients with a CALLY index <6.5 (40% vs. 76%; *p* < 0.001). Furthermore, patients with advanced age (*p* = 0.002), poor ECOG PS, anemia, elevated LDH, CRP, β2M, and hypoalbuminemia (all *p* < 0.001) also had significantly worse OS. Tumor-associated histological characteristics, including subtype and biomarker expression, were not significantly related to OS, except for Ki-67 >70% (*p* = 0.021). Among clinical factors, bulky disease was associated with worse OS, whereas EN involvement and CS alone did not significantly affect survival. High IPI, poor R-IPI, and high-risk NCCN-IPI were all strongly predictive of inferior OS (*p* < 0.001) (Table 2, Figure 2).

In the multivariate Cox regression model, CALLY index <6.5 remained an independent predictor of worse OS (HR 2.2, 95% CI 1.2–4.0, *p* = 0.011), along with Ki-67 >70% (HR 1.6 95% CI 1.0–2.7, *p* = 0.046).

**Figure 3:**
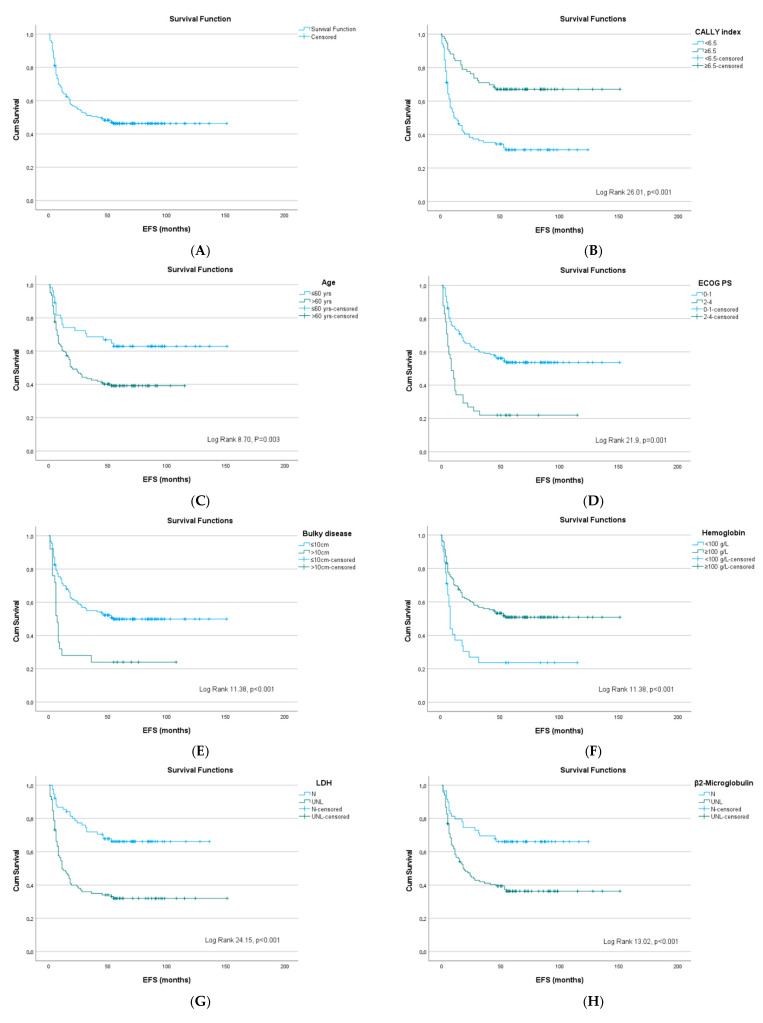

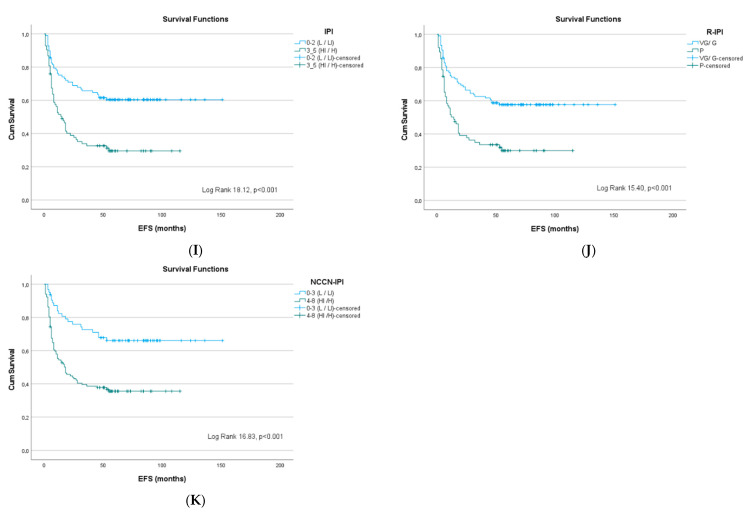
Kaplan–Meier curves for event-free survival in patients with DLBCL, NOS: (**A**) entire cohort; stratified by (**B**) CALLY index, (**C**) age, (**D**) ECOG PS, (**E**) bulky disease, (**F**) hemoglobin level, (**G**) lactate dehydrogenase, (**H**) β2-microglobulin, (**I**) IPI, (**J**) R-IPI, and (**K**) NCCN-IPI.

The estimated three- and five-year EFS rates were 51.0% and 46.0%, respectively. Shorter EFS was significantly associated with age, poor ECOG PS, anemia, elevated LDH, CRP, β2M, and hypoalbuminemia (all *p* < 0.05). Other baseline laboratory parameters, comorbidities, and DLBCL -related clinical and histological markers, were not significantly correlated with EFS. Among clinical factors, bulky disease was associated with worse EFS. Similarly to OS, patients with high IPI (Log-rank 18.1, *p* < 0.001), poor R-IPI (Log-rank 15.4, *p* < 0.016), high NCCN-IPI (Log-rank 16.8, *p* < 0.000), and CALLY index <6.5 had significantly worse EFS (Log-rank 26.0, *p* < 0.001). In the multivariate analysis, only CALLY index <6.5 (HR 1.90, 95% CI 1.11–3.26, *p* = 0.019) was identified as the sole independent predictor of inferior EFS (Table 3, Figure 3).

### 3.3. Predictors of Response to Treatment

The ORR to first-line treatment for the entire cohort was 70.6%. Patients with a low CALLY index demonstrated a significantly lower ORR compared to those with a high index (59.6% vs. 85.5%; *p* < 0.001). Of the 180 patients, 70 (38.9%) were classified as relapsed/refractory (R/R). The incidence of R/R status was significantly higher in the low CALLY group compared to the high CALLY group (52.9% vs. 19.7%; *p* < 0.001).

#### 3.3.1. ORR Analysis

Univariate logistic regression identified several factors associated with a lower ORR, including a low CALLY index, bulky disease, anemia (Hb < 100 g/L), elevated AMC, and high-risk prognostic scores (Table 4). To ensure model stability and avoid data redundancy, the multivariate model was constructed using consolidated markers (NCCN-IPI and CALLY index) alongside independent clinical variables. In this analysis, anemia (OR: 3.0, 95% CI: 1.2–7.5, *p* = 0.019) and elevated AMC (OR: 2.7, 95% CI: 1.3–5.8; *p* = 0.008) remained independent predictors of poor response.

#### 3.3.2. R/R Status Analysis

Univariate analysis identified low CALLY index, NCCN-IPI, bulky disease, anemia, and elevated β2M as significant predictors for R/R status (Table 4). In the final multivariate model, a low CALLY index remained a robust independent predictor of R/R status, with patients in the low-index group having over a three-fold higher risk of relapse or refractory disease (OR: 3.2, 95% CI: 1.5–7.0; *p* = 0.004). Additionally, anemia was identified as an independent risk factor for R/R status. (OR: 2.8, 95% CI: 1.0–7.6; *p* = 0.048).

## 4. Discussion

Currently, the IPI, R-IPI, and NCCN-IPI are the most widely used clinical tools for stratifying patients with DLBCL, guiding treatment decisions, and estimating survival. However, their reliance on static clinical variables, such as age, disease stage, ECOG PS, and serum LDH, limits their capacity to predict individual patient outcomes, especially in the era of precision medicine [53,54]. This drives the search for additional biomarkers that better reflect the biological mechanisms of underlying disease progression and response to treatment. In recent years, advances in multi-omics, gene-expression profiling, and immune-microenvironment characterization have yielded several promising molecular prognostic signatures [55,56,57]. Yet despite their potential, these molecularly driven tools are often time-consuming, costly, and impractical for routine clinical implementation, highlighting the need for simplified, biologically informed but clinically feasible prognostic approaches.

One particularly promising domain in biomarker development lies in the intersection of host inflammatory response, immune competence, and nutritional status, which together influence tumor behavior and treatment outcomes. Systemic inflammation, as evidenced by elevated acute phase reactants such as CRP, has been universally linked with increased cancer risk [58] and adverse prognosis in many malignancies [59], including lymphomas [60]. Elevated CRP reflects activation of the innate immune system, often driven by tumor-derived cytokines such as interleukin-6 (IL-6), which in turn promotes tumor proliferation, survival, and the creation of an immunosuppressive microenvironment [23,61,62]. On the nutritional front, serum albumin is a well-established prognostic biomarker across numerous cancers, including lymphomas [63,64]. Hypoalbuminemia, often secondary to inflammation-induced catabolism, indicates diminished physiological reserves and impaired immune competence. Because albumin also exerts anti-inflammatory and antioxidant effects, low albumin levels may reflect a composite state of nutritional deficiency and pro-tumor inflammation [25,65]. In this way, hypoalbuminemia serves as a surrogate indicator of both systemic stress and host vulnerability to disease progression and treatment toxicity [66,67]. The absolute lymphocyte count (ALC) is another compelling marker that captures the state of host immune competence. Lymphocyte subsets play pivotal roles in tumor immune surveillance. Accordingly, lymphopenia has been associated with poorer chemotherapy response, weaker anti-tumor immunity, and inferior OS [22,27,68,69]. In the context of DLBCL, lymphopenia may reflect systemic immunosuppression and a diminished capacity for anti-tumor immune control [70,71,72].

In our multicenter cohort of 180 newly diagnosed DLBCL, NOS patients, those with a low CALLY index (<6.5), higher CRP, and low serum albumin, had significantly inferior OS and EFS, while ALC had no prognostic impact. Notably, the CALLY index was the only variable to remain an independent prognostic factor in multivariate analysis. Our findings underscore that reliance on a single inflammation marker fails to capture the complexity of host–tumor interplay, limiting predictive performance. By integrating these parameters, the CALLY index offers a broader, more powerful risk estimate than any single marker alone.

Our results demonstrate that a low CALLY index is significantly associated with a wide range of adverse baseline laboratory and clinical features, including anemia, elevated LDH and β_2_M, impaired ECOG PS, and bulky disease, while, importantly, showing no apparent influence from patient comorbidities, suggesting that the CALLY index primarily reflects tumor burden and a more aggressive lymphoma course. Several traditional adverse prognostic factors, such as EN involvement, advanced Ann Arbor stage, COO subtype, and expression of immunohistochemical markers (except Ki-67), did not correlate with the CALLY index or predict OS or EFS in our cohort. This echoes previous observations that many standard histopathological and clinical markers have diminished predictive value in the rituximab era [73,74,75,76] and underscores the imperative for biomarkers that integrate both tumor biology and host response.

The low CALLY index was significantly correlated with higher risk strata according to IPI, R-IPI, and NCCN-IPI. While IPI and its derivatives have long served as pillars of risk stratification, their ability to discriminate in the ICT era is limited, particularly for patients with a less than 50% chance of long-term survival [77,78].

We recently presented preliminary data at a scientific congress, demonstrating that the CALLY index is a promising prognostic tool in DLBCL, with a strong association with both OS and EFS [35]. These initial observations were subsequently corroborated by Pinar et al., who reported concordant findings in a single-center study including 112 patients with DLBCL from Turkey [36].

In the present study, we extend these earlier findings by providing a comprehensive and more granular analysis of our cohort. Notably, we demonstrate that the CALLY index not only retains its prognostic significance but also emerges as an independent and robust predictor of both OS and EFS, outperforming traditional prognostic models. Importantly, the CALLY index enabled clinically meaningful risk discrimination: more than 50% of patients in the low-CALLY group exhibited resistance to frontline treatment or experienced lymphoma recurrence, whereas fewer than 20% of patients in the high-CALLY group had these adverse outcomes.

Crucially, stratification using the IPI, R-IPI, or NCCN-IPI did not reliably identify the subgroup at the highest risk of treatment failure or relapse. These findings highlight a key novel contribution of our study—the ability of the CALLY index to refine risk assessment beyond established prognostic indices. Our data indicate that the CALLY index serves as a valuable complementary tool, particularly by identifying high-risk R/R patients who may otherwise remain insufficiently recognized by standard indices, thereby offering potential implications for treatment stratification and clinical decision-making.

## 5. Conclusions

The present study provides the first comprehensive evidence supporting the prognostic relevance of the CALLY index in DLBCL, NOS not only for survival, but also for identifying patients at increased risk of treatment resistance, relapse, and inferior outcomes. Based on routinely available laboratory parameters, namely, C-reactive protein, serum albumin, and absolute lymphocyte count, the CALLY index represents a practical, accessible, and cost-effective tool that may complement established prognostic models in clinical practice. These findings must be considered alongside the retrospective design and modest sample size. Nevertheless, the multicenter design, the minimization of biological heterogeneity by restricting the cohort to patients with newly diagnosed DLBCL, NOS, and the uniform administration of frontline rituximab-based ICT, which ensures comparable treatment exposure and minimizes the confounding impact of differences in treatment intensity, represent key strengths of this study.

Prospective, multicenter studies in larger patient populations are needed to validate the prognostic utility of the CALLY index and clarify its role in guiding risk-adapted therapeutic strategies. With further validation, the CALLY index could advance individualized prognostic assessment and management in DLBCL.

## Figures and Tables

**Table 1:** Clinicopathological characteristics, laboratory parameters, histopathology, prognostic scores, treatment response, and outcomes of patients with DLBCL, NOS stratified by CALLY index.

Category/Variable	Overall N = 180 (*n*, %)	CALLY < 6.5 N = 104 (57.8) (*n*, %)	CALLY ≥ 6.5 N = 76 (42.2) (*n*, %)	*p*-Value
**Demographics**				
Age: >60 years	125 (69.4)	78 (75.0)	47 (61.8)	0.058
Sex: Female/Male	101 (56.1)/ 79 (43.9)	66 (63.5)/ 38 (36.5)	35 (46.1)/ 41 (53.9)	**0.020**
**Performance/Comorbidities**				
ECOG: PS ≥ 2	41 (22.8)	35 (33.7)	6 (7.9)	**<0.001**
Comorbidities: Yes	143 (79.4)	84 (80.8)	59 (77.6)	0.607
CIRS: >6	28 (15.6)	19 (18.3)	9 (11.8)	0.240
**Clinical Characteristics**				
Ann Arbor CS: III–IV	114 (63.3)	82 (78.8)	32 (42.1)	<0.001
Bulky disease: >10 cm	25 (13.9)	23 (22.1)	2 (2.6)	<0.001
EN: Yes	81 (45.0)	49 (47.1)	32 (42.1)	0.505
**Laboratory Parameters**				
Hb: <100 g/L	31 (17.2)	27 (26.0)	4 (5.3)	**<0.001**
ALC: <1 × 10^9^/L	51 (28.3)	40 (38.5)	11 (14.5)	**<0.001**
AMC: >0.63 × 10^9^/L	65 (36.1)	42 (40.4)	23 (35.4)	0.163
Platelets: <157 × 10^9^/L	22 (12.2)	15 (14.4)	7 (9.2)	0.292
LDH: ULN	104 (57.8)	77 (74.0)	27 (35.5)	**<0.001**
Albumin: <35 g/L	48 (26.7)	45 (43.3)	3 (3.9)	**<0.001**
Β2M: ULN	121 (67.2)	89 (85.6)	32 (42.1)	**<0.001**
CRP: ULN	121 (67.2)	100 (96.2)	21 (27.6)	**<0.001**
**Histopathology/Immunohistochemistry**				
COO: Non-GCB/GCB	120 (66.7)/ 60 (33.3)	69 (66.3)/ 35 (33.7)	51 (67.1)/ 25 (32.9)	0.915
BCL2: Positive	118 (65.6)	69 (66.3)	49 (64.5)	0.794
BCL6: Positive	130 (72.2)	79 (76.0)	51 (67.1)	0.190
Ki-67: >70%	111 (61.7)	67 (64.4)	44 (57.9)	0.374
**Prognostic Scores**				
IPI: 0–2 (L/LI) vs. 3–5 (HI/H)	97 (53.9)/ 83 (46.1)	36 (34.6)/ 68 (65.4)	61 (80.3)/ 15 (19.7)	**<0.001**
R-IPI: VG/G vs. Poor	105 (58.3)/ 75 (41.7)	39 (37.5)/ 65 (62.5)	66 (86.8)/ 10 (13.2)	**<0.001**
NCCN-IPI: 0–3 (L/LI) vs. 4–8 (HI/H)	63 (35.0)/ 117 (65.0)	21 (20.2)/ 83 (79.8)	42 (55.3)/ 34 (44.7)	**<0.001**
**Treatment Response and Outcome**				
ORR: CR + PR vs. SD + PD	127 (70.6)/ 53 (29.4)	62 (59.6)/ 42 (40.4)	65 (85.5)/ 11 (14.5)	**<0.001**
Relapse: Yes	17/127 (13.4)	13/62 (21.0)	4/65 (6.2)	**0.014**
R/R: Yes	70 (38.9)	55 (52.9)	15 (19.7)	**<0.001**
Alive/Dead	101 (56.1)/ 79 (43.9)	43 (41.3)/ 61 (58.7)	58 (76.3)/ 18 (23.7)	**<0.001**
EFS endpoint	95 (52.8)	70 (67.3)	25 (32.9)	**<0.001**
Non-DLBCL-related death	18/79 (22.8)	11/61 (18.0)	7/18 (38.9)	0.064

Abbreviations: ECOG PS—Eastern Cooperative Oncology Group performance status; CIRS—Cumulative Illness Rating Scale; CS—clinical stage; EN—extranodal involvement; Hb—hemoglobin; ALC—absolute lymphocyte count; AMC—absolute monocyte count; LDH—lactate dehydrogenase; β2M—β2-microglobulin; CRP—C-reactive protein; ULN—upper limit of normal; COO—cell of origin; GCB—germinal center B-cell-like; IPI—International Prognostic Index; L—low; LI—low-intermediate; HI—high-intermediate; H—high; R-IPI—revised International Prognostic Index; VG—very good; G—good; NCCN-IPI—National Comprehensive Cancer Network International Prognostic Index; ORR—overall response rate; CR—complete remission; PR—partial remission; SD—stable disease; PD—progressive disease; R/R—relapsed/refractory; EFS—event free survival; DLBCL—Diffuse large B-cell lymphoma. Statistically significant values are highlighted in bold.

**Table 2:** Overall survival with univariate and multivariate Cox regression.

Category/ Variable		Mean OS ± SE (95% CI)	Median OS (95% CI)	3-Year OS (%)	5-Year OS (%)	Log Rank	*p*-Value (Log Rank)	Univariate HR (95% CI)	*p*-Value (Uni)	Multivariate HR (95% CI)	*p*-Value (Multi)
CALLY	≥6.5	119.3 ± 6.6 (106.4–132.1)	NR *	81.6	75.5	24.8	**<0.001**	1.0 3.50 (2.1–5.9)	**<0.001**	1.0 2.2 (1.2–4.0)	**0.011**
	<6.5	59.3 ± 5.7 (48.1–70.5)	24.0 (9.1–38.9)	43.7	39.6						
**Demographics**											
Age (years)	≤60	114.1 ± 8.1 (98.2–130.0)	NR	76.0	71.5	9.7	**0.002**	1.0 2.35 (1.3–4.1)	**0.003**	-	-
	>60	62.3 ± 4.6 (53.2–71.4)	46.0 (NE **)	53.0	47.9						
Sex	Female	87.4 ± 6.9 (73.8–101.0)	NR	57.0	53.2	0.2	0.616	-	-	-	-
	Male	84.7 ± 6.8 (71.3–98.2)	NR	63.8	57.2						
**Performance/Comorbidities**											
ECOG PS	<2	103.8 ± 5.5 (93.0–114.6)	NR	70.1	64.2	34.5	**<0.001**	1.0 3.47 (2.2–5.5)	**<0.001**	-	-
	≥2	35.39 ± 7.1 (21.4–49.4)	12.0 (7.0–16.9)	24.4	24.4						
Comorbidities	No	109.5 ± 10.5 (88.9–130.1)	NR	75.2	75.2	3.4	0.066	-	-	-	-
	Yes	77.3 ± 5.2 (67.1–87.4)	NR	56.1	51.2						
CIRS	≤6	93.5 ± 5.5 (82.7–104.4)	NR	61.9	57.7	2.6	0.110	-	-	-	-
	>6	59.4 ± 10.7 (38.3–80.5)	24.0 (21.6–66.1)	50.0	38.7						
**Clinical characteristics**											
Ann Arbor CS	I-II	103.5 ± 8.3 (87.4–119.7)	NR	70.8	65.4	3.1	0.078	-	-	-	-
	III-IV	75.4 ± 5.7 (64.2–86.6)	48.0 (NE)	54.0	51.4						
Bulky > 10 cm	No	95.2 ± 5.4 (84.5–105.8)	NR	64.1	58.3	8.4	**0.004**	1.0 2.24 (1.3–3.9)	**0.004**	1.0 1.2 (0.7–2.19)	0.466
	Yes	42.7 ± 9.5 (23.9–61.4)	12.0 (2.5–21.5)	33.7	33.7						
EN	No	74.9 ± 6.4 (61.8–87.2)	NR	57.6	52.4	0.6	0.473	-	-	-	-
	Yes	93.5 ± 6.8 (80.0–107.0)	NR	61.9	57.3						
**Laboratory parameters**											
Hb (g/L)	≥100	97.2 ± 5.5 (86.3–108.0)	NR	64.0	60.1	11.8	**<0.001**	1.0 2.31 (1.4–3.8)	**<0.001**	1.0 1.4 (0.8–2.42)	0.177
	<100	44.3 ± 8.7 (27.2–61.3)	16.0 (4.51–27.5)	40.4	30.3						
ALC (×10^9^/L)	≥1	95.5 ± 5.9 (83.9–107.2)	NR	64.6	58.5	3.5	0.061	-	-	-	-
	<1	69.7 ± 8.7 (51.8–86.4)	30.0 (NE)	48.2	46.1						
AMC (×10^9^/L)	≤0.63	88.4 ± 5.6 (77.5–99.2)	NR	67.1	59.4	3.1	0.076	-	-	-	-
	>0.63	78.8 ± 8.6 (61.9–95.6)	35.0 (NE)	47.7	47.7						
Platelets (×10^9^/L)	≥157	83.6 ± 4.8 (74.2–93.0)	NR	53.1	48.3	1.3	0.246	-	-	-	-
	<157	78.66 ± 15.3 (46.6–108.7)	46.0 (NE)	60.9	55.9						
LDH (U/L)	N	107.7 ± 5.8 (96.3–119.2)	NR	81.3	75.0	23.7	**<0.001**	1.0 3.36 (2.0–5.7)	**<0.001**	-	-
	ULN	68.8 ± 6.7 (55.6–82.1)	25.0 (10.7–39.3)	44.3	40.4						
Albumin (g/L)	≥35	102.0 ± 5.7 (90.7–113.2)	NR	69.1	63.1	18.1	**<0.001**	1.0 2.5 (1.6–3.9)	**<0.001**	-	-
	<35	45.9 ± 7.1 (31.9–60.0)	15.0 (3.7–26.3)	35.4	33.3						
β2M (mg/L)	N	100.2 ± 6.5 (87.5–112.9)	NR	81.4	73.8	12.4	**<0.001**	1.0 2.7 (1.5–4.7)	**<0.001**	1.0 1.6 (0.8–2.9)	0.159
	ULN	76.9 ± 6.3 (64.5–89.3)	36.0 (NE)	49.3	45.7						
CRP (mg/L)	N	108.1 ± 6.6 (95.2–120.9)	NR	83.1	75.2	15.7	**<0.001**	1.0 3.0 (1.7–5.4)	**<0.001**	-	-
	ULN	75.4 ± 6.5 (62.9–87.8)	35.0 (14.2–55.8)	48.4	44.9						
**Histopathology/Immunohistochemistry**											
COO	Non-GCB	84.7 ± 7.1 (72.4–97.0)	NR	57.2	50.8	2.1	0.152	-	-	-	-
	GCB	84.6 ± 6.3 (70.6–98.5)	NR	65.7	63.8						
BCL2	Negative	94.4 ± 8.7 (77.3–111.4)	NR	60.3	57.9	0.4	0.500	-	-	-	-
	Positive	79.8 ± 5.7 (68.7–90.9)	NR	59.9	53.6						
BCL6	Negative	71.7 ± 8.2 (55.7–87.7)	60.0 (NE)	56.0	48.9	0.7	0.400	-	-	-	-
	Positive	93.1 ± 6.0 (81.3–104.9)	NR	61.6	57.5						
Ki-67	≤70%	104.1 ± 7.7 (88.9–119.3)	NR	69.4	64.3	5.3	**0.021**	1.0 1.7 (1.1–2.8)	**0.023**	1.0 1.6 (1.01–2.72)	**0.046**
	>70%	70.4 ± 5.6 (59.4–81.4)	48.0 (NE)	54.0	49.2						
**Prognostic scores**											
IPI	0–2 (L/LI)	110.2 ± 6.3 (97.8–122.7)	NR	74.9	69.1	18.5	**<0.001**	1.0 2.6 (1.6–4.1)	**<0.001**	─ *^a^*	─ *^a^*
	3–5 (HI/H)	52.8 ± 5.6 (41.9–63.7)	24.0 (14.3–33.7)	42.8	39.0						
R-IPI	VG/G	106.7 ± 6.3 (94.5–119.0)	NR	71.9	66.6	15.6	**<0.001**	1.0 2.4 (1.5–3.7)	**<0.001**	─ *^a^*	─ *^a^*
	Poor	53.0 ± 5.9 (41.4–64.5)	24.0 (12.5–35.5)	43.4	39.1						
NCCN-IPI	0–3 (L/LI)	120.4 ± 6.9 (106.8–133.9)	NR	83.8	75.1	18.2	**<0.001**	1.0 3.2 (1.8–5.6)	**<0.001**	1.0 1.8 (1.0–3.3)	0.058
	4–8 (HI/H)	57.9 ± 4.8 (48.5–67.3)	27.0 (3.3–51.0)	47.1	44.4						
**Treatment response/Outcome**											
ORR	CR + PR	120.0 ± 4.9 (110.3–129.7)	NR	81.4	75.2	155.3	**<0.001**	1.0 12.0 (7.4–19.5)	**<0.001**	-	-
	SD + PD	13.7 ± 2.2 (9.6–17.7)	8.0 (6.2–9.8)	9.4	7.5						
Relapse	No	94.1 ± 5.4 (83.4–104.8)	NR	61.8	58.8	4.0	**0.047**	1.0 1.7 (1.0–3.1)	0.05	-	-
	Yes	40.1 ± 6.0 (28.3–51.7)	28.0 (17.6–38.4)	44.4	22.2						
Non-DLBCL-related death	No	25.4 ± 4.2 (16.8–33.5)	18.0 (13.9–22.1)	22.2	5.6	8.6	**0.003**	1.0 2.17 (1.2–3.8)	**0.007**	-	-
	Yes	13.52 ± 1.4 (10.7–16.2)	10 (7.3–12.2)	8.2	0						

* NR: Median survival not reached (cumulative survival remained above 50% throughout follow-up). ** NE: 95% CI not estimable due to insufficient events at the end of the follow-up period. ^a^ Variable excluded from the multivariable model due to high multicollinearity with NCCN-IPI. Abbreviations: ECOG PS—Eastern Cooperative Oncology Group performance status; CIRS—Cumulative Illness Rating Scale; CS—clinical stage; EN—extranodal involvement; Hb—hemoglobin; ALC—absolute lymphocyte count; AMC—absolute monocyte count; LDH—lactate dehydrogenase; β2M—β2-Microglobulin; CRP—C-reactive protein; N—normal; ULN—upper limit of normal; COO—cell of origin; GCB—germinal center B-cell-like; IPI—International Prognostic Index; L—low; LI—low-intermediate; HI—high-intermediate; H—high; R-IPI—revised International Prognostic Index; VG—very good; G—good; NCCN-IPI—National Comprehensive Cancer Network International Prognostic Index; ORR—overall response rate; CR—complete remission; PR—partial remission; SD—stable disease; PD—progressive disease; DLBCL—Diffuse large B-cell lymphoma; OS—overall survival; SE—Standard Error. Statistically significant values are highlighted in bold.

**Table 3:** Event-free survival with univariate and multivariate Cox regression.

Variable		Mean EFS ± SE (95% CI)	Median EFS (95% CI)	3-Year EFS (%)	5-Year EFS (%)	Log Rank	*p*-Value (Log Rank)	Univariate HR (95% CI)	*p*-Value (Uni)	Multivariate HR (95% CI)	*p*-Value (Multi)
CALLY	≥6.5	107.2 ± 7.2 (93.0–121.4)	NR *	71.1	67.1	26.0	**<0.001**	1.0 3.1 (1.9–4.8)	**<0.001**	1.0 1.9 (1.1–3.3)	**0.019**
	<6.5	46.3 ± 5.3 (35.9–56.6)	12.0 (5.2–18.7)	35.3	30.9						
**Demographics**											
Age (years)	≤60	101.2 ± 8.9 (83.6–118.7)	NR	68.7	62.9	8.7	**0.003**	1.0 2.1 (1.3–3.4)	**0.004**	-	-
	>60	52.4 ± 4.6 (43.4–61.4)	20.0 (11.4–28.6)	42.6	39.2						
Sex	Female	75.9 ± 6.9 (62.2–89.6)	32.0 (NE **)	49.1	45.7	0.1	0.826	-	-	-	-
	Male	71.3 ± 7.0 (57.5–85.1)	45.0 (NE)	52.5	47.0						
**Performance/Comorbidities**											
ECOG PS	<2	88.2 ± 5.9 (76.7–99.7)	NR	59.1	53.6	21.9	**<0.001**	1.0 2.6 (1.7–4.1)	**<0.001**	-	-
	≥2	31.7 ± 7.0 (18.1–45.4)	8.0 (4.2–11.7)	22.0	22.0						
Comorbidities	No	98.2 ± 11.2 (76.3–120.1)	NR	67.0	61.3	3.6	0.057	-	-	-	-
	Yes	65.2 ± 5.2 (55.0–75.4)	27.0 (9.4–44.6)	46.4	42.5						
CIRS	≤6	80.1 ± 5.7 (68.9–91.3)	53.0 (NE)	52.2	48.5	2.3	0.130	-	-	-	-
	>6	37.3 ± 6.8 (24.0–50.6)	19.0 (6.0–32.0)	42.1	34.4						
**Clinical characteristics**											
Ann Arbor CS	I–II	87.1 ± 8.6 (70.1–104.1)	NR	58.7	54.1	1.6	0.209	-	-	-	-
	III–IV	64.7 ± 5.8 (53.3–76.1)	27.0 (8.4–45.6)	45.9	41.6						
Bulky > 10 cm	No	82.7 ± 5.6 (71.8–93.7)	55.0 (NE)	54.9	49.9	11.4	**<0.001**	1.0 2.3 (1.4–3.8)	**<0.001**	1.0 1.4 (0.8–2.4)	0.177
	Yes	31.3 ± 8.7 (14.3–48.4)	7.0 (5.4–8.6)	24.0	24.0						
EN	No	63.6 ± 6.6 (50.7–76.6)	46.0 (NE)	48.0	45.2	0.4	0.546	-	-	-	-
	Yes	79.5 ± 6.9 (65.8–93.2)	27.0 (NE)	52.8	47.2						
**Laboratory parameters**											
Hb (g/L)	≥100	83.9 ± 5.7 (72.8–95.1)	NR	56.0	50.9	11.4	**<0.001**	1.0 2.2 (1.4–3.5)	<0.001	1.0 1.5 (0.9–2.6)	0.104
	<100	33.9 ± 8.3 (17.6–50.3)	8.0 (6.7–9.3)	23.7	23.7						
ALC (×10^9^/L)	≥1	81.8 ± 6.1 (69.7–93.8)	53.0 (NE)	53.1	49.8	2.8	0.093	-	-	-	-
	<1	59.2 ± 8.7 (42.3–76.2)	22.0 (14.6–50.6)	44.3	37.6						
AMC (×10^9^/L)	≤0.63	78.0 ± 5.7 (66.7–89.3)	NR	57.6	51.9	5.2	**0.022**	1.0 1.6 (1.1–2.4)	**<0.001**	1.0 1.5 (1.0–2.3)	0.064
	>0.63	61.8 ± 8.4 (45.1–78.5)	14.0 (6.1–21.9)	38.5	6.5						
Platelets (×10^9^/L)	<157	63.7 ± 15.2 (33.9–93.5)	9.0 (0.0–39.9)	43.3	37.9	1.5	0.217	-	-	-	-
	≥157	71.7 ± 4.9 (62.0–81.4)	46.0 (NE)	51.7	47.5						
LDH (U/L)	N	96.7 ± 6.5 (83.9–109.4)	NR	71.9	66.1	24.1	**<0.001**	1.0 2.9 (1.8–4.7)	**<0.001**	-	-
	ULN	55.6 ± 6.5 (42.8–68.4)	11.0 (4.9–17.1)	35.1	32.0						
Albumin (g/L)	≥35	86.8 ± 6.0 (75.0–98.7)	NR	57.7	52.7	13.1	**<0.001**	1.0 2.1 (1.4–3.2)	**<0.001**	-	-
	<35	39.9 ± 7.1 (26.0–53.7)	8.0 (4.9–11.0)	31.3	29.0						
β2-M (mg/L)	N	86.9 ± 6.8 (73.6–100.3)	NR	69.5	66.1	13.0	**<0.001**	1.0 2.4 (1.5–3.9)	**<0.001**	1.0 1.4 (0.8–2.4)	0.231
	ULN	63.1 ± 6.3 (50.9–75.3)	18.0 (9.1–26.9)	41.2	36.4						
CRP (mg/L)	N	100.8 ± 7.0 (87.0–114.4)	NR	74.6	69.5	20.2	**<0.001**	1.0 3.1 (1.8–5.1)	**<0.001**	-	-
	ULN	60.1 ± 6.2 (48.0–72.3)	16.0 (10.4–21.6)	38.7	34.9						
**Histopathology/Immunohistochemistry**											
COO	Non-GCB	73.6 ± 6.3 (61.3–86.1)	28.0 (20.0–57.8)	48.3	43.8	0.7	0.411	-	-	-	-
	GCB	56.9 ± 5.7 (45.7–68.1)	NR	55.4	51.8						
BCL2	Negative	85.5 ± 8.9 (68.1–102.9)	NR	57.6	52.2	1.4	0.238	-	-	-	-
	Positive	66.1 ± 5.7 (54.8–77.3)	31.0 (3.1–58.9)	46.9	43.2						
BCL6	Negative	65.2 ± 8.3 (48.9–81.4)	25.0 (NE)	50.0	45.6	0.2	0.896	-	-	-	-
	Positive	77.6 ± 6.1 (65.5–89.6)	45.0 (NE)	50.8	46.6						
Ki-67	≤70%	86.2 ± 8.2 (70.1–102.3)	NR	59.2	51.7	2.7	0.101	-	-	-	-
	>70%	61.4 ± 5.6 (50.4–72.5)	25.0 (11.0–50.3)	45.2	43.0						
**Prognostic scores**											
IPI	0–2 (L/LI)	97.2 ± 6.8 (83.8–110.7)	NR	65.7	60.4	18.1	**<0.001**	1.0 2.4 (1.6–3.6)	**<0.001**	─ *^a^*	─ *^a^*
	3–5 (HI/H)	42.3 ± 5.4 (31.8–52.9)	15.0 (9.2–20.8)	32.6	29.5						
R-IPI	VG/G	93.5 ± 6.6 (80.5–106.5)	NR	62.6	57.7	15.4	**<0.001**	1.0 2.2 (1.4–3.3)	**<0.001**	─ *^a^*	─ *^a^*
	Poor	42.6 ± 5.7 (31.4–53.8)	14.0 (8.0–20.0)	33.5	30.0						
NCCN-IPI	0–3 (L/LI)	106 ± 8.0 (90.6–122.0)	NR	72.7	66.1	16.8	**<0.001**	1.0 2.6 (1.6–4.3)	**<0.001**	1.0 1.6 (1.0–2.7)	0.072
	4–8 (HI/H)	48.3 ± 4.7 (39.1–57.6)	18.0 (8.5–27.5)	38.7	35.6						
**Treatment response/Outcome**											
ORR	(CR + PR)	107.1 ± 5.5 (96.2–118.0)	NR	71.9	65.8	207.7	**<0.001**	1.0 20.3 (12.1–34.2)	**<0.001**	-	-
	(SD + PD)	5.54 ± 0.6 (4.3–6.8)	5.0 (4.0–6.0)	0.0	0.0						
Relapse	No	83.4 ± 5.6 (72.4–94.3)	NR	53.8	51.8	8.7	**0.003**	1.0 2.1 (1.3–3.5)	**0.005**	-	-
	Yes	23.3 ± 3.6 (16.3–30.3)	17.0 (14.2–19.8)	22.2	0.0						
Non-DLBCL-related death	No	21.7 ± 4.0 (13.9–29.5)	18.0 (9.7–26.2)	16.7	5.6	17.2	**<0.001**	1.0 3.1 (1.7–5.6)	**<0.001**	-	-
	Yes	7.6 ± 0.92 (8.2–13.4)	5.0 (3.9–6.1)	1.6	0.0						

* NR: Median survival not reached (cumulative survival remained above 50% throughout follow-up). ** NE: 95% CI not estimable due to insufficient events at the end of the follow-up period. *^a^* Variable excluded from the multivariable model due to high multicollinearity with NCCN-IPI. Abbreviations: ECOG PS—Eastern Cooperative Oncology Group performance status; CIRS—Cumulative Illness Rating Scale; CS—clinical stage; EN—extranodal involvement; Hb—hemoglobin; ALC—absolute lymphocyte count; AMC—absolute monocyte count; LDH—lactate dehydrogenase; β2M—β2-Microglobulin; CRP—C-reactive protein; N—normal; ULN—upper limit of normal; COO—cell of origin; GCB—germinal center B-cell-like; IPI—International Prognostic Index; L—low; LI—low-intermediate; HI—high-intermediate; H—high; R-IPI—revised International Prognostic Index; VG—very good; G—good; NCCN-IPI—National Comprehensive Cancer Network International Prognostic Index; ORR—overall response rate; CR—complete remission; PR—partial remission; SD—stable disease; PD—progressive disease; DLBCL—Diffuse large B-cell lymphoma; EFS—event free survival; SE—Standard Error. Statistically significant values are highlighted in bold.

**Table 4:** Logistic regression for overall response rate and relapsed/refractory cases.

Variable/Category		ORR (N = 127)		R/R (N = 70)	
		OR (95% CI)	*p* Value	OR (95% CI)	*p* Value
CALLY	<6.5 vs. ≥6.5	4.0 (1.9–8.5)	**<0.001**	5.7 (2.8–11.5)	**< 0.001**
**Demographics**					
Age (years)	>60 vs. ≤60	2.4 (1.1–5.1)	**0.031**	2.6 (1.3–5.2)	**0.009**
Sex	Female vs. Male	1.03 (0.5–2.0)	0.931	1.09 (0.6–2.0)	0.786
**Performance/Comorbidities**					
ECOG PS	≥2 vs. <2	4.6 (2.2–9.7)	**<0.001**	4.9 (2.1–11.4)	**<0.001**
Comorbidities	Yes vs. No	1.7 (0.7–3.9)	0.245	1.7 (0.8–3.7)	0.169
CIRS	>6 vs. ≤6	1.1 (0.39–2.3)	0.912	1.3 (0.5–3.2)	0.626
**Clinical Characteristics**					
Ann Arbor CS	III–IV vs. I–II	1.1 (0.5–2.1)	0.883	1.5 (0.8–2.8)	0.245
Bulky > 10 cm	Yes vs. No	4.6 (1.9–11.1)	**0.001**	4.3 (1.5–12.3)	**0.008**
EN	Yes vs. No	1.4 (0.7–2.7)	0.301	1.2 (0.6–2.2)	0.659
**Laboratory Parameters**					
Hb (g/L)	<100 vs. ≥100	3.2 (1.5–7.2)	**0.004**	4.5 (1.8–11.4)	**0.001**
ALC (×10^9^/L)	<1 vs. ≥1	1.7 (0.8–3.3)	0.150	1.6 (0.8–3.2)	0.198
AMC (×10^9^/L)	>0.63 vs. ≤0.63	2.2 (1.1–4.2)	**0.021**	1.8 (0.9–3.6)	0.071
Platelets (×10^9^/L)	<157 vs. ≥157	1.8 (0.7–4.5)	0.212	1.4 (0.5–3.9)	0.465
LDH (U/L)	ULN vs. N	6.5 (2.8–14.8)	**<0.001**	8.1 (3.8–17.3)	**<0.001**
Albumin (g/L)	<35 vs. ≥35	4.60 (2.3–9.3)	**<0.001**	3.5 (1.7–7.3)	**<0.001**
β2M (mg/L)	ULN vs. N	1.7 (0.8–3.6)	0.130	3.3 (1.6–6.6)	**<0.001**
CRP (mg/L)	ULN vs. N	4.6 (1.9–10.9)	**0.001**	6.1 (2.7–13.5)	**<0.001**
**Histopathology/Immunohistochemistry**					
COO	Non GCB vs. GCB	1.2 (0.6–2.4)	0.563	1.2 (0.6–2.4)	0.571
BCL2	Positive vs. Negative	1.7 (0.8–3.4)	0.145	1.4 (0.7–2.7)	0.301
BCL6	Positive vs. Negative	1.2 (0.6–2.4)	0.641	1.1 (0.6–2.3)	0.739
MUM1	Positive vs. Negative	1.4 (0.7–2.7)	0.361	1.2 (0.6–2.3)	0.660
CD47	Positive vs. Negative	1.3 (0.5–3.3)	0.575	1.2 (0.5–2.8)	0.708
Ki67 (%)	>70 vs. ≤70%	1.9 (0.9–3.7)	0.076	1.8 (1.0–3.6)	0.069
**Prognostic Scores**					
IPI	HI/H vs. L/LI	2.8 (1.5–5.5)	**0.002**	3.7 (1.9–7.3)	**<0.001**
R-IPI	Poor vs. Very Good/Good	2.7 (1.4–5.1)	**0.004**	3.4 (1.7–6.5)	**<0.001**
NCCN-IPI	HI/H vs. L/LI	4.3 (1.9–9.8)	**0.001**	4.7 (2.2–9.8)	**<0.001**
**Multivariate analysis of factors predicting ORR and R/R**					
**Variable/Category**		**ORR (N = 127)**		**R/R (N = 70)**	
		**OR (95% CI)**	***p *** **value**	**OR (95% CI)**	***p *** **value**
CALLY	<6.5 vs. ≥6.5	1.8 (0.8–4.3)	0.161	3.2 (1.5–7.0)	**0.004**
Bulky >10 cm	Yes vs. No	2.4 (0.9–6.1)	0.073	2.4 (0.8–7.4)	0.132
Hb (g/L)	<100 vs. ≥100	3.0 (1.2–7.5)	**0.019**	2.8 (1.0–7.6)	**0.048**
AMC (×10^9^/L)	>0.63 vs. ≤0.63	2.7 (1.3–5.8)	**0.008**	-	-
NCCN-IPI	HI/H vs. L/LI	2.4 (1.0–5.8)	0.063	2.2 (1.0–4.7)	0.057

Abbreviations: ECOG PS—Eastern Cooperative Oncology Group performance status; CIRS—Cumulative Illness Rating Scale; CS—clinical stage; EN—extranodal involvement; Hb—hemoglobin; ALC—absolute lymphocyte count; AMC—absolute monocyte count; LDH—lactate dehydrogenase; β2M—β2-Microglobulin; CRP—C-reactive protein; N—normal; ULN—upper limit of normal; COO—cell of origin; GCB—germinal center B-cell-like; IPI—International Prognostic Index; L—low; LI—low-intermediate; HI—high-intermediate; H—high; R-IPI—revised International Prognostic Index; VG—very good; G—good; NCCN-IPI—National Comprehensive Cancer Network International Prognostic Index; ORR—overall response rate; R/R—relapsed/refractory. Statistically significant values are highlighted in bold.

## Data Availability

The data that support the findings of this study are available on request from the corresponding author.

## Supplementary Materials

The following supporting information can be downloaded at: https://www.mdpi.com/article/10.3390/cancers18050846/s1, Table S1: AUC values of CALLY index ROC curve analysis at multiple time points.

## Abbreviations

The following abbreviations are used in this manuscript:

ALC	Absolute lymphocyte count
AMC	Absolute monocyte count
AUC	Area under the curve
β2M	Beta2- microglobulin
CALLY	C-reactive protein–albumin–lymphocyte
CI	Confidence interval
CIRS	Cumulative Illness Rating Scale
COO	Cell of origin
CR	Complete remission
CRP	C-reactive protein
DLBCL	Diffuse large B cell lymphoma
ECOG	Eastern Cooperative Oncology Group
EFS	Event free survival
EN	Extranodal
F/M	Female to male ratio
GCB	Germinal center B cell like
Hb	Hemoglobin
HR	Hazard ratio
ICT	Immunochemotherapy
IL-6	Interleukin-6
IPI	International Prognostic Index
IQR	Interquartile range
LDH	Lactate dehydrogenase
NCCN-IPI	National Comprehensive Cancer Network International Prognostic Index
NHL	Non Hodgkin lymphoma
NOS	Not otherwise specified
OR	Odds ratio
ORR	Overall response rate
OS	Overall survival
PR	Partial remission
PS	Performance status
R-CHOP	Rituximab cyclophosphamide doxorubicin vincristine and prednisone
R-IPI	Revised International Prognostic Index
R/R	Relapsed or refractory
ROC	Receiver operating characteristic
ULN	Upper limit of normal
WHO HAEM5	World Health Organization Classification of Haematolymphoid Tumours 5th edition
