# Supplementary material for: Prognostic Value of the CALLY Index in Diffuse Large B-Cell Lymphoma: Linking Inflammation, Nutrition, and Tumor Biology
Source: Cancers (Basel). 2026 Mar 5;18(5):846. doi: 10.3390/cancers18050846 (PMC12984221; doi:10.3390/cancers18050846)
Supplement: Supplementary file 1 [file cancers-18-00846-s001.zip › cancers-4131593-supplementary.pdf]

**Supplementary Table S1**. AUC values of CALLY index ROC curve analysis at multiple time points.

| Time Point | AUC | 95% CI | *p*-value |
|---|---|---|---|
| 2-Year | 0.733 | (0.657–0.809) | <0.001 |
| 3-Year | 0.755 | (0.682–0.828) | <0.001 |
| 5-Year | 0.733 | (0.659–0.808) | <0.001 |
| 10-Year | 0.733 | (0.659–0.808) | <0.001 |
